# Epidemiology and risk factors associated with gout control among adult Asians: a real-world retrospective cohort study

**DOI:** 10.3389/fmed.2023.1253839

**Published:** 2023-09-07

**Authors:** Prawira Oka, Wei Ming Chong, Ding Xuan Ng, Wai Keong Aau, Ngiap Chuan Tan

**Affiliations:** ^1^SingHealth Polyclinics, Singapore, Singapore; ^2^SingHealth-Duke NUS Family Medicine Academic Clinical Program, Singapore, Singapore; ^3^Lee Kong Chian School of Medicine, Singapore, Singapore

**Keywords:** allopurinol, family medicine, gout, primary care, cohort

## Abstract

**Background:**

Gout is associated with significant morbidity and mortality, yet suboptimal gout control remains a problem globally. Identifying the risk factors associated with poor gout control among patients in primary care allows targeted interventions to improve their clinical management. This study aimed to determine the prevalence of poor gout control and its associated demographic and clinical factors among urbanized community-dwelling Asian patients.

**Methods:**

This retrospective study was based on data extracted from the electronic medical records of 8 public primary care clinics in Singapore. Patients with a diagnostic code of gout who had 2 or more visits between 1st January 2018 and 31st December 2019 were included in the analysis. Data extracted included: demographics, anthropological measurements, comorbidities, serum uric acid levels and medication prescription. A patient is defined to have poor gout control if they suffer two or more acute gout attacks within a year. Chi-Squared test was used for categorical parameters. For continuous variables, univariate logistic regression analysis was first performed. Significant factors (*p* ≤ 0.1) were then included in the logistics regression model to account for confounders.

**Results:**

A total of 7,970 patients and 24,624 visits were included in the analysis. The prevalence of poorly controlled gout was 28.2% (*n* = 2,244/7,970); only 46.3% of them (*n* = 1,039/2,244) were prescribed allopurinol and 13.4% (*n* = 301/2,244) were taking doses ≥300 mg. Using logistic regression, factors associated with poor gout control were: male gender [adjusted OR (AOR) =1.66, *p* < 0.001], Malay ethnicity (AOR = 1.27, *p* = 0.007), congestive heart failure (AOR = 1.64, *p* = 0.037). Patients prescribed allopurinol (AOR = 1.52, *p* < 0.001), NSAIDs (AOR = 2.76, *p* < 0.001) and corticosteroids (AOR = 2.83, *p* < 0.001) were more likely to have poorly-controlled gout.

**Conclusion:**

Nearly 30% of patients had poor gout. Interventions should focus on male and Malay patients and those with congestive cardiac failure.

## Background

Gout is a common inflammatory arthritis rising in prevalence both globally and locally, with a prevalence of 4.1% among Singaporean Chinese (1, 2). During an acute gout flare, patients can experience excruciating pain within the first 24 h. Chronic poorly controlled gout can result in tophi and joint deformities (3). Aside from increased morbidity, the prospective Health Professionals Follow-up study revealed an elevated all-cause mortality rate in men with gout (4).

Despite the significant potential burden of illness, gout control remains suboptimal across the world. Evidence has shown that achieving serum uric acid (SUA) levels ≤360 μmol/L leads to the dissolution of crystal deposits, tophi resolution, and reduces the frequency of acute gout attacks (5, 6). However, a large proportion of patients globally have not attained SUA targets (7–9). Similarly, a local study found that only 22.3% of patients achieved the SUA target (10). These studies highlight the significant potential for improved gout control.

Suboptimal gout control is multifactorial, involving both modifiable and non-modifiable risk factors (11). Previous studies have assessed the relationship between gout control, intensity and adherence to urate-lowering therapy (ULT) (12, 13). Apart from pharmacological therapy, demographic and clinical risk factors are potential factors that can influence gout control. Male gender and increased affluence have been associated with gout (1). Additionally, individuals with chronic kidney disease (CKD) and congestive heart failure (CHF) have a six-fold and four-fold risk of gout, respectively (14). While these studies have identified epidemiological factors associated with gout, few have quantified their impact on gout control in community-dwelling patients (11, 15, 16).

Recognizing the factors associated with poor gout control facilitates the identification of at-risk patients and the development of targeted interventions to improve their health outcomes (11). Therefore, this study aims to determine the demographic and clinical risk factors associated with poor gout control among Asian adults who are managed in primary healthcare clinics, where most of them are treated in urbanized Singapore.

## Methods

Singapore’s population is multi-ethnic comprising mainly Chinese (75.9%), Malay (15%), and Indian (7.5%) ethnicities (17). The local residents can access healthcare services in private and public primary care clinics. Patient registry is not mandated at any primary care clinic as an individual can walk in readily for consultation during clinic hours.

SingHealth Polyclinics (SHP) delivers comprehensive public primary care services to the populace across the eastern region of the island-state (18). Its network of eight polyclinics managed more than 2.5 million patient attendances for both acute and chronic ailments in 2019.

### Study design

A retrospective cohort study was conducted on patients with gout who consulted any of the eight SHP clinics between 1st January 2018 and 31st December 2019.

### Participants

**Figure 1:**
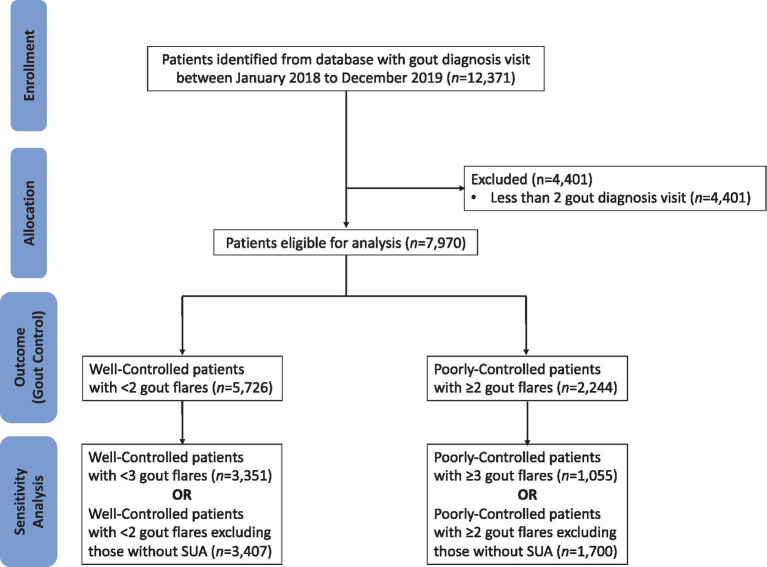
CONSORT diagram depicting patients screened before analysis.

The CONSORT diagram outlining how the patients are screened prior to analysis is shown in Figure 1. Patients who visited SHP two or more times with clinician-diagnosed gout were included in the study population. For patients with gout, clinicians enter the International Classification of Diseases (ICD-10) code of “gout” into the Sunrise Clinical Manager electronic medical records (EMR). Patients with less than two consultations for gout within the study period were excluded from the study to exclude those without any care continuity at any of the study sites.

### Data extraction

In SHP, doctors utilize the Sunrise Clinical Manager^®^ (SCM^®^) electronic medical record system to administer and document clinical care. Sociodemographic and financial status is captured separately in the Outpatient Administrative System (OAS) which primarily deals with patients’ appointments and billing. Data from SCM and OAS are stored on separate databases. The patients’ healthcare data is routinely extracted from these databases and transformed into its desired actionable format through the ETL (Extract, Transform, and Load) database function. Thereafter, the transformed data from multiple healthcare transactional systems were integrated in a single enterprise data repository known as the Electronic Health Intelligence System (eHINTS).

A research informatics staff from the Research Department in SHP extracted the data via eHINTS (16). In accordance with the institutional data governance and protection policy, an externally appointed trusted third party (TTP) assisted to de-identify the data. Finally, the TTP ported the de-identified data over to the research team *via* a secure file transfer protocol for analysis.

Data extracted included socio-demographic factors, anthropological measurements, comorbidities and allopurinol prescription. Data on socio-demographics including gender, age, ethnicity, smoking and medical subsidy status was collected. Patients were considered to be on medical subsidy if they received any financial assistance during the study period. The mean BMI of each subject during the study period was categorized as underweight (BMI < 18.5), normal (18.5 ≤ BMI < 23), overweight (23 ≤ BMI < 27.5) or obese (BMI ≥ 27.5) based on the Ministry of Health (MOH) Clinical Practice Guidelines (CPG) on obesity (19). Associated comorbid conditions including congestive heart failure, chronic kidney disease, diabetes mellitus, hyperlipidemia, hypertension and ischemic heart disease were obtained from the EMR.

### Definition of poorly-controlled gout

Patients were deemed to have poorly-controlled gout if they suffered two or more gout attacks within 12 months. A gout attack was defined as an SHP clinic visit with an EMR diagnosis of “gout” and colchicine prescription not prescribed as prophylaxis or standby. Colchicine prescription was classified as prophylaxis or standby should the electronic prescription records reflect a comment stating this indication. To compare for variation in associated factors, sensitivity analyses was conducted with a revised definition of poorly-controlled gout (three or more gout attacks within 12 months).

### Statistical analysis

Data were presented in frequencies and percentages for categorical demographics and mean ± standard deviation (SD) for continuous parameters. Categorical parameters were compared for association with gout control using the Chi-Squared test. Univariate and bivariate logistic regression analyses were performed to explore the continuous and categorical variables associated with gout control, respectively. Significant factors (*p* ≤ 0.1) from these analyses were included in the logistics regression model to account for confounders. Adjusted odds ratio (AOR) and confidence intervals were presented for factors associated with poor gout control. To assess the stability and reliability of the results, sensitivity analyses were conducted. The analysis involved adjusting the definition for poorly-controlled gout and excluding patients without SUA to ensure the robustness of results. Missing values were imputed with either the mean or median depending on the normality of distribution. All analysis was carried out using IBM SPSS version 26.0 and R version 3.5.2. Statistical significance was set at *p* ≤ 0.05.

### List of covariates included in the multivariate analysis

- Demographic: age, gender, ethnicity.

- Lifestyle: smoking status and BMI.

- Comorbidities: chronic kidney disease, congestive heart failure, type 2 diabetes mellitus, hyperlipidemia, hypertension and ischemic heart disease.

- Medications: allopurinol, NSAIDs, thiazide diuretics, furosemide, prednisolone, losartan and amlodipine.

## Results

The baseline characteristics of the study population are shown in Table 1. A total of 7,970 patients with gout visited SingHealth Polyclinics (SHP) between 2018 and 2019 with an EMR diagnosis of gout were included in the analysis. These patients had a total of 24,624 consultations for gout within the study period. The majority of patients were males (83.6%), Chinese (76.8%) with a mean age of 61.7 ± 14.2 years. Overall, 28.2% of patients had poorly-controlled gout. The most common urate lowering therapy was allopurinol (*n* = 3,176), less were prescribed febuxostat (*n* = 9) and probenecid (*n* = 78). The overall median index SUA was 456 μmol/L, with significantly higher SUA among patients with poorly-controlled gout (489 vs. 438 μmol/L).

**Table 1:** Baseline characteristics of the study population (*n* = 7,970).

	Total	Well-controlled gout	Poorly-controlled gout*	*p*-value
Total	7,970 (100)	5,726 (71.8)	2,244 (28.2)	
Total gout visits	24,624 (100)	15,846 (64.4)	8,778 (35.6)	
Age [mean (SD)]	61.7 (14.2)	63.6 (13.5)	56.8 (14.9)	<0.001
Gender				<0.001
Male	6,666 (83.6)	4,608 (80.5)	2,058 (91.7)	
Female	1,304 (16.4)	1,118 (19.5)	186 (8.3)	
Ethnicity				<0.001
Chinese	6,123 (76.8)	4,493 (78.5)	1,630 (72.6)	
Malay	1,206 (15.2)	771 (13.5)	435 (19.4)	
Indian	297 (3.7)	224 (3.9)	73 (3.3)	
Others	344 (4.3)	238 (4.2)	106 (4.7)	
Smoking status				0.150
Non-smoker	7,131 (89.5)	5,142 (89.8)	1,989 (88.6)	
Ex-smoker (stopped >6 months)	74 (0.9)	56 (1.0)	18 (0.8)	
Smoker	765 (9.6)	528 (9.2)	237 (10.6)	
Medical subsidy				0.786
No	4,575 (57.4)	3,281 (57.3)	1,294 (57.7)	
Yes	3,395 (42.6)	2,445 (42.7)	950 (42.3)	
BMI				0.104
Underweight	80 (1.2)	67 (1.4)	13 (0.8)	
Normal	986 (15.3)	760 (15.6)	226 (14.2)	
Overweight	2,590 (40.1)	1,955 (40.2)	635 (39.8)	
Obese	2,807 (43.4)	2,087 (42.9)	720 (45.2)	
Congestive heart failure				0.029
No	7,853 (98.5)	5,653 (98.7)	2,200 (98.0)	
Yes	117 (1.5)	73 (1.3)	44 (2.0)	
Chronic kidney disease				0.001
No (eGFR ≥60)	6,638 (83.3)	4,720 (82.4)	1,918 (85.5)	
Stage 3,4 or 5 eGFR <60	1,332 (16.7)	1,006 (17.6)	326 (14.5)	
Type 2 diabetes mellitus				<0.001
No	5,600 (70.3)	3,824 (66.8)	1,776 (79.1)	
Yes	2,370 (29.7)	1,902 (33.2)	468 (20.9)	
Hyperlipidemia				<0.001
No	2,099 (26.3)	1,208 (21.1)	891 (39.7)	
Yes	5,871 (73.7)	4,518 (78.9)	1,353 (60.3)	
Hypertension				<0.001
No	1,647 (20.7)	891 (15.6)	756 (33.7)	
Yes	6,323 (79.3)	4,835 (84.4)	1,488 (66.3)	
Ischemic heart disease				<0.001
No	6,613 (83.0)	4,679 (81.7)	1,934 (86.2)	
Yes	1,357 (17.0)	1,047 (18.3)	310 (13.8)	
Allopurinol				<0.001
No	4,794 (60.2)	3,589 (62.7)	1,205 (53.7)	
Yes	3,176 (39.8)	2,137 (37.3)	1,039 (46.3)	
NSAIDs				<0.001
No	3,954 (49.6)	3,329 (58.1)	625 (27.9)	
Yes	4,016 (50.4)	2,397 (41.9)	1,619 (72.1)	
Thiazides				0.023
No	7,303 (91.6)	5,221 (91.2)	2,082 (92.8)	
Yes	667 (8.4)	505 (8.8)	162 (7.2)	
Furosemide				0.204
No	7,626 (95.7)	5,468 (95.5)	2,158 (96.2)	
Yes	344 (4.3)	258 (4.5)	86 (3.8)	
Prednisolone				<0.001
No	6,481 (81.3)	4,920 (85.9)	1,561 (69.6)	
Yes	1,489 (18.7)	806 (14.1)	683 (30.4)	
Losartan				<0.001
No	6,242 (78.3)	4,337 (75.7)	1,905 (84.9)	
Yes	1,728 (21.7)	1,389 (24.3)	339 (15.1)	
Amlodipine				<0.001
No	5,437 (68.2)	3,710 (64.8)	1,727 (77)	
Yes	2,533 (31.8)	2,016 (35.2)	517 (23)	
Febuxostat				0.999
No	7,961 (99.9)	5,720 (99.9)	2,241 (99.9)	
Yes	9 (0.1)	6 (0.1)	3 (0.1)	
Probenecid				0.251
No	7,892 (99.0)	5,675 (99.1)	2,217 (98.8)	
Yes	78 (1.0)	51 (0.9)	27 (1.2)	
Index SUA μmol/L (median [IQR])	456 [380–523]	438 [363–508]	489 [424–547]	<0.001
Final SUA μmol/L (median [IQR])	435 [363–501]	423 [355–490]	458 [388–521]	<0.001

*Poorly-controlled gout defined as ≥ 2 gout attacks in 12 months.

The factors associated with poor gout control are presented in Table 2. Younger, males and patients of Malay ethnicity along with patients with higher SUA were more likely to have poorly-controlled gout. Patients prescribed allopurinol, NSAIDs and corticosteroids had increased odds of poorly-controlled gout. Patients with diabetes, hyperlipidemia and hypertension were less likely to have poorly-controlled gout. Those on losartan and amlodipine also had reduced odds for poorly-controlled gout.

**Table 2:** Factors associated with poor gout control.

	Poorly-controlled (≥2 gout attacks) (*n* = 2,244)		Poorly-controlled (≥3 gout attacks) (*n* = 1,055)	
Characteristics	Adjusted OR (95%CI)	*P*-value	Adjusted OR (95%CI)	*P*-value
Age	0.99 (0.99–1.00)	0.011	0.99 (0.99–1.01)	0.426
Gender				
Female	Ref	–	Ref	–
Male	1.66 (1.37–2.02)	<0.001	1.51 (1.15–2.02)	0.004
Ethnicity				
Chinese	Ref	–	Ref	–
Malay	1.27 (1.07–1.5)	0.007	1.49 (1.18–1.88)	0.001
Indian	0.92 (0.66–1.26)	0.612	0.95 (0.60–1.47)	0.829
Others	0.99 (0.73–1.35)	0.985	1.24 (0.81–1.84)	0.306
Smoking status				
Non-smoker	Ref	–	Ref	–
Ex-smoker	0.73 (0.39–1.3)	0.299	1.04 (0.43–2.28)	0.928
Smoker	1.03 (0.84–1.26)	0.754	0.91 (0.68–1.21)	0.530
BMI				
Underweight	0.81 (0.41–1.49)	0.515	1.24 (0.47–2.86)	0.637
Normal	Ref	–	Ref	–
Overweight	0.99 (0.82–1.2)	0.924	1.17 (0.88–1.55)	0.281
Obese	0.9 (0.73–1.09)	0.278	0.96 (0.72–1.29)	0.791
Comorbidities				
Chronic kidney disease				
No (eGFR ≥60)	Ref	–	Ref	–
Stage 3,4 or 5 (eGFR<60)	1.09 (0.92–1.3)	0.307	0.89 (0.70–1.12)	0.329
Congestive heart failure	1.64 (1.02–2.6)	0.037	1.54 (0.80–2.88)	0.186
Type 2 diabetes mellitus	0.82 (0.71–0.95)	0.007	0.76 (0.62–0.94)	0.010
Hyperlipidemia	0.79 (0.67–0.92)	0.003	0.87 (0.69–1.09)	0.228
Hypertension	0.75 (0.62–0.91)	0.003	0.70 (0.54–0.91)	0.008
Ischemic heart				
Disease	1.01 (0.85–1.19)	0.954	1.14 (0.90–1.44)	0.271
Medications				
Allopurinol	1.52 (1.34–1.72)	<0.001	1.60 (1.34–1.92)	<0.001
NSAIDs	2.76 (2.42–3.14)	<0.001	2.57 (2.12–3.12)	<0.001
Thiazides	1.19 (0.96–1.47)	0.111	1.09 (0.81–1.45)	0.571
Prednisolone	2.83 (2.44–3.28)	<0.001	2.36 (1.94–2.87)	<0.001
Losartan	0.84 (0.72–0.98)	0.027	0.80 (0.64–0.99)	0.043
Amlodipine	0.82 (0.71–0.93)	0.003	0.69 (0.57–0.85)	<0.001
Laboratory				
Index SUA (per 10 μmol/L)	1.03 (1.02–1.03)	<0.001	1.02 (1.01–1.03)	<0.001

Sensitivity analysis for 3 or more gout attacks revealed that all factors remained significant except gender and patients with congestive heart failure or hyperlipidemia. For factors that remained significant, there was negligible change on the direction of association. However, a notable increase in the coefficients of odds ratio was observed for ethnic Malay patients, signifying a stronger association.

The factors associated with poor gout control, excluding patients without SUA, are presented in Table 3. Sensitivity analysis was done excluding patients without a SUA laboratory result. A total of 36% (*n* = 2,863) of patients without a SUA result were excluded from this analysis. Logistic regression revealed that males, Malays and patients with higher SUA had significantly higher odds for poorly-controlled gout. Additionally, patients prescribed allopurinol, NSAIDs and corticosteroids had increased odds for poorly-controlled gout.

**Table 3:** Factors associated with poor gout control excluding patients without SUA (*N* = 5,107).

	Poorly-controlled (≥2 gout attacks)	
Characteristics	Adjusted OR (95%CI)	*P*-value
Age	0.99 (0.99–1.00)	0.091
Gender		
Female	Ref	–
Male	1.31 (1.04–1.65)	0.022
Ethnicity		
Chinese	Ref	–
Malay	1.29 (1.05–1.58)	0.014
Indian	1.21 (0.82–1.76)	0.339
Others	0.94 (0.65–1.33)	0.716
Smoking status		
Non-smoker	Ref	–
Ex-smoker	0.84 (0.43–1.58)	0.598
Smoker	1.09 (0.86–1.37)	0.457
BMI		
Underweight	0.55 (0.20–1.30)	0.207
Normal	Ref	–
Overweight	1.03 (0.82–1.29)	0.814
Obese	0.87 (0.68–1.10)	0.245
Comorbidities		
Chronic kidney disease		
No (eGFR ≥60)	Ref	–
Stage 3,4 or 5 (eGFR <60)	1.01 (0.82–1.22)	0.977
Congestive heart failure	1.35 (0.71–2.53)	0.349
Type 2 diabetes mellitus	0.95 (0.80–1.13)	0.550
Hyperlipidemia	0.82 (0.68–0.98)	0.031
Hypertension	0.83 (0.67–1.04)	0.106
Ischemic heart disease	1.03 (0.84–1.25)	0.790
Medications		
Allopurinol	1.17 (1.01–1.35)	0.034
NSAIDs	2.46 (2.11–2.86)	<0.001
Thiazides	1.09 (0.85–1.39)	0.479
Prednisolone	2.58 (2.18–3.07)	<0.001
Losartan	0.85 (0.72–1.02)	0.076
Amlodipine	0.84 (0.72–0.98)	0.028
Laboratory		
Index SUA (per 10 μmol/L)	1.03 (1.02–1.04)	<0.001

**Figure 2:**
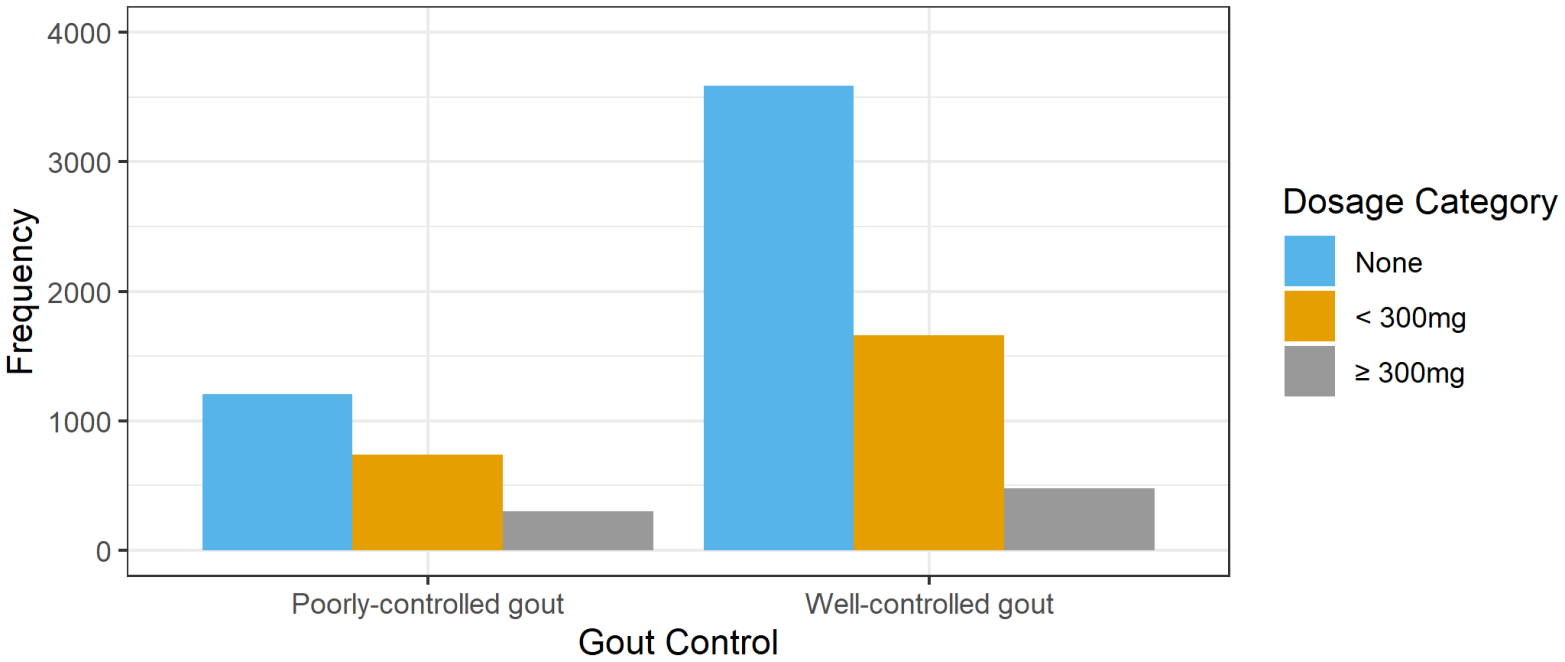
Prescribed allopurinol dose in patients with poorly-controlled vs. well-controlled gout.

The prescribed allopurinol doses among patients with poorly-controlled and well-controlled gout is shown in Figure 2. Nearly two-thirds (62.7%, *n* = 3,589) of patients with well-controlled gout were not treated with allopurinol. More than half (53.6%, *n* = 1,205) of patients with poorly-controlled gout were not prescribed allopurinol; 13.4% (*n* = 301) were on allopurinol doses ≥300 mg.

## Discussion

### Demographic profile of patients with gout

The findings were consistent with known gout epidemiology, predominantly affecting Chinese males, as Chinese is the majority ethnic group in Singapore (1). More than a quarter of patients had poorly-controlled gout, greater than the 11 and 22% reported in earlier American and local studies, respectively (7, 8).

Males were more likely to have poorly-controlled gout, consistent with literature reporting lower SUA levels and less frequent gout attacks among female patients (11). In females, estrogen is postulated to have a protective effect through enhancing renal uric acid excretion (20, 21). Local reports show higher rates of adherence to ULT among female patients with gout, contributing to better gout control (12).

Malay patients were at increased risk of having poorly-controlled gout. This contrasts with a previous local study that revealed an increased risk of poorly-controlled gout in Chinese patients (22). MacFarlane and Kim. alluded to dietary triggers such as alcohol intake that predispose to hyperuricemia (16). Nevertheless, as Muslims, the vast majority of Malays in Singapore are forbidden to consume alcohol (17). Aside from abstaining from alcohol and pork, Malay individuals’ diets do not differ significantly from the other ethnicities. Contrary to the preconception that gout is a “disease of the kings,” prior studies have shown lower socioeconomic status to be associated with poorly-controlled gout (23). Based on the 2020 Census, the median household income from work per household member is lowest among Malay individuals at SGD$1,594 compared with Chinese (SGD$2,603) and Indian individuals (SGD$2,521) (24).

Ultimately, the mechanism that facilitates increased incidence of poorly-controlled gout in Malay patients remains unclear and requires further exploration.

### Comorbidities associated with poor gout control

Patients with gout had a higher prevalence of comorbidities. Hypertension, hyperlipidemia, and diabetes prevalence among the study patients was 79.3, 73.7, and 29.7% respectively; this contrasts with 21.5, 33.6, and 8.6%, respectively, among the general population in Singapore (25). Patients with type-2 diabetes mellitus, hyperlipidemia and hypertension were less likely to have poorly-controlled gout. Medications like losartan and SGLT-2 inhibitors, which are often prescribed for patients with hypertension and diabetes, are suggested to have a protective role against gout flares (26, 27). Furthermore, earlier local studies had reported superior ULT adherence among patients with comorbidities, consistent with similar findings among patients with other chronic diseases such as renal impairment and asthma (12). Patients with other chronic conditions are regularly reviewed by primary care clinicians for their disease control and counseled for their diet and medication adherence (10, 12). They are likely to take medications daily to treat the multi-morbidities, in which allopurinol if prescribed, is one of them.

In contrast, patients with congestive heart failure were more likely to have poorly-controlled gout. Frequently, a loop or thiazide diuretic is prescribed to manage their fluid status, which elevates their serum uric acid level and aggravates their risk of acute gout (28).

### Medications associated with poor gout control

Patients prescribed NSAIDs and corticosteroids were more likely to have poorly- controlled gout. The 2020 American College of Rheumatology guidelines recommend that aside from colchicine, NSAIDs and corticosteroids as agents to treat patients suffering from an acute gout flare. Hence, patients prescribed these agents would be more likely to suffer an acute gout flare and be consequently classified as having poorly-controlled gout (29).

Chronic medications such as losartan and amlodipine were associated with a reduced probability of poorly-controlled gout. Losartan is an established uricosuric agent shown to reduce serum uric levels and improve gout control (30). Juraschek et al. found amlodipine was associated with a 37% reduced risk of gout (31). It was postulated that calcium channel blockers promote uricosuria via URAT1 transporter inhibition, thereby reducing hyperuricemia and consequent gout flares (32).

### Allopurinol prescription

Patients on allopurinol were one and a half times more likely to have poorly-controlled gout. The definition of poorly-controlled gout adopted can explain this finding as patients suffering two or more flares a year should be started on allopurinol. Concerningly, allopurinol was only prescribed in approximately half of patients with poorly-controlled gout, placing these patients at increased morbidity and mortality risks (4). An Australian study revealed a similarly low allopurinol prescription of 37.1% (33). Some patients only consult a doctor when they develop acute gouty arthritis but default review to initiate ULT. In primary care, allopurinol is the most commonly prescribed ULT for patients with uncontrolled gout.

The low allopurinol prescription is likely due to therapeutic inertia from both physicians and patients (34). Physicians are often concerned about the risk of allopurinol-induced severe cutaneous adverse reactions (SCAR) (35). The risk of SCAR is further heightened in the local population where the majority ethnic group is Chinese. Literature has revealed that Han Chinese are at increased risk of possessing the HLA-B*5801 allele, which predisposes them to the risk of SCAR (36). Testing for the allele is available albeit costly, with a local study concluding that HLA-B*5801-guided ULT selection was not cost-effective based on a threshold of US$50,000 per quality-adjusted life year (37). The health authorities recognized this concern and released an advisory to all medical professionals that routine HLA-B*1501 testing is not recommended before initiating a patient on allopurinol (38). The advisory also states that healthcare providers may consider HLA-B*5801 testing individuals with an elevated risk for SCAR, such as those with renal impairment or advanced age (38). Nonetheless, the worry about iatrogenic SCAR remains, probably due to the severity of its consequences, even though only approximately three out of a thousand individuals are at risk of developing SCAR (38).

In addition to the genetic predisposition for SCAR, reduced renal function is a further risk factor for SCAR (38). Consequently, optimal control of gout is often not achieved among patients with reduced renal function (39). Despite recommendations by the American College of Rheumatology (ACR) that patients with renal impairment should be initiated on lower doses of 50 mg of allopurinol daily, therapeutic inertia persists due to physicians lacking awareness and familiarity with these recommendations (29, 40, 41).

Aside from allopurinol initiation, only 14.3% of patients with poorly-controlled gout on allopurinol were given doses of ≥300 mg. A prospective study showed that 70% of patients required an allopurinol dose of 300 mg/day to achieve the therapeutic target, with the remainder requiring even higher doses (42). Similarly, a retrospective study in Malaysia reported that the mean allopurinol daily doses required were 290 and 369 mg in individuals with non-tophaceous and tophaceous gout, respectively, (43). The suboptimal dosing may stem from reduced awareness and familiarity with treatment targets. A Chinese study revealed that only 54.6% of general practitioners were aware of the treatment target, with only 5.6% possessing a good understanding of gout (44). Perry et al. postulated that general practitioners may be less likely to access published guidelines or updates presented on rheumatology-specific platforms, which invariably affects the quality of care (35). This is especially salient as the majority of patients with gout are treated in primary care. In Singapore, an appropriate care guide on gout management was jointly developed and published in 2019 by the Agency of Care Effectiveness, Ministry of Health, College of Family Physicians and College of Physicians with representation from the Chapter of Renal Medicine Physicians and Rheumatologists (45). Nevertheless, the results from this study suggest a pertinent need to mitigate the therapeutic inertia of primary care physicians. In an Asia-Pacific update on gout, Paul and James urged primary care physicians to optimize the currently available treatment options to improve patient care (46).

### Colchicine prescription

Colchicine was prescribed in 41.8% of the visits and was provided for acute flare (82.5%), prophylaxis (11.3%) or standby (6.2%). Prophylaxis is indicated when patients with gout undergo ULT initiation or titration (29). Given that only 3.77% (301/7,970) of patients with poorly-controlled gout were receiving allopurinol doses under 300 mg, the rate of colchicine prescribed as prophylaxis appears proportional as these individuals could be undergoing dose titration. The rate of colchicine prescribed as prophylaxis is reassuring given that George et al. found nearly three-quarters of patients were inappropriately prescribed colchicine prophylaxis despite not being on ULT or undergoing dose titration (47).

### Strength and limitations

Data collated from multiple polyclinics in this primary care study over time is a strength, as the results reflect a good representation of the clinical outcomes of patients with gout in a highly urbanized community. Primary care physicians should leverage the study results to enhance their treatment plan for their patients with gout and reduce their suffering from acute exacerbation of affected joints.

The revelation that Malay ethnicity is a risk factor represents another discovery in this study. Unlike earlier studies that revealed Chinese as a demographic risk for gout, the Malays are the minority in the multi-ethnic population in Singapore. It can pave the way for further research to understand the ethnic variations in gout control across the communities in Singapore and beyond in the Asia Pacific region.

Retrospective research is limited by the invariable missing data across the observation period. Verification of the data from the EMR is also challenging. The absence of a specific diagnosis of acute gout exacerbation to define the subset of the study population with poor gout control is another study limitation. Additionally, a prior study among Swiss physicians showed that only 66% of primary care physicians demonstrated sufficient knowledge to manage gout in comparison with 93% of rheumatologists (48). Hence, individuals with non-gouty arthritis could have been incorrectly diagnosed as the study relied on diagnosis being made by non-rheumatology specialists.

Locally, colchicine is mainly prescribed to manage acute gout exacerbations instead of as prophylaxis. This trend was also seen in our study, with 82.5% of colchicine prescribed for an acute gout flare. However, colchicine was also prescribed as prophylaxis or as standby. In these patients, it is difficult to ascertain if they ultimately consumed the colchicine for an acute flare thereby underestimating the prevalence of patients with poorly-controlled gout. Furthermore, patients experiencing a gout attack could be prescribed non-steroidal anti-inflammatory drugs (NSAIDs) or steroids instead of colchicine. Consequently, the prevalence of poorly-controlled gout might be underestimated. In this study, NSAIDs and steroids were not used to define an acute gout flare as these drugs are commonly prescribed for other conditions including non-gouty arthritis and asthma exacerbations. Finally, the data from the EMR did not include dietary triggers such as alcohol intake and purine-rich food consumption.

## Conclusion

The study identified Malay, male gender and those with congestive cardiac failure as demographic and comorbid risk factors associated with poor gout control. Nearly 30% of patients had poor gout control. Overall, the low allopurinol prescription rates and suboptimal dosing in patients with poorly-controlled gout are of concern. Primary care physicians need to optimize ULT for their patients to regain and sustain gout control.

## Data availability statement

The raw data supporting the conclusions of this article will be made available by the authors, without undue reservation.

## Ethics statement

Ethical approval was not required for the study involving humans in accordance with the local legislation and institutional requirements. Written informed consent to participate in this study was not required from the participants or the participants’ legal guardians/next of kin in accordance with the national legislation and the institutional requirements.

## Author contributions

PO: Writing – original draft, Writing – review & editing. WC: Conceptualization, Writing – original draft, Writing – review & editing. DN: Formal analysis, Writing – review & editing. WA: Data curation, Formal analysis, Writing – review & editing. NT: Conceptualization, Supervision, Writing – original draft, Writing – review & editing.

## Funding

This study was supported in-kind (research expertise) by the Research Department in SingHealth Polyclinics. The publication cost is supported by SingHealth Polyclinics – Centre Grant CG21APR3006 (NMRC/CG3/001/2022-SHP).

## Conflict of interest

The authors declare that the research was conducted in the absence of any commercial or financial relationships that could be construed as a potential conflict of interest.

## Publisher’s note

All claims expressed in this article are solely those of the authors and do not necessarily represent those of their affiliated organizations, or those of the publisher, the editors and the reviewers. Any product that may be evaluated in this article, or claim that may be made by its manufacturer, is not guaranteed or endorsed by the publisher.

## Abbreviations

ACR: American College of Rheumatology
CHF: congestive heart failure
CKD: chronic kidney disease
CPG: clinical practice guidelines
eHINTS: Electronic Health Intelligence System
ETL: extract, transform and load
EMR: electronic medical records
ICD-10: International Classification of Diseases
MOH: Ministry of Health
OAS: Outpatient Administrative System
NSAIDs: non-steroidal anti-inflammatory drugs
SCAR: severe cutaneous adverse reactions
SCM^®^: Sunrise Clinical Manager^®^
SD: standard deviation
SHP: SingHealth Polyclinics
SUA: serum uric acid
TTP: trusted third party
ULT: urate-lowering therapy
